# Why overlearned sequences are special: distinct neural networks for ordinal sequences

**DOI:** 10.3389/fnhum.2012.00328

**Published:** 2012-12-20

**Authors:** Vani Pariyadath, Mark H. Plitt, Sara J. Churchill, David M. Eagleman

**Affiliations:** ^1^Department of Neuroscience, Baylor College of MedicineHouston, TX, USA; ^2^Department of Psychiatry, Baylor College of MedicineHouston, TX, USA

**Keywords:** overlearned sequence, synesthesia, fMRI, semantic dementia, language, right hemisphere, predictability

## Abstract

Several observations suggest that overlearned ordinal categories (e.g., letters, numbers, weekdays, months) are processed differently than non-ordinal categories in the brain. In synesthesia, for example, anomalous perceptual experiences are most often triggered by members of ordinal categories (Rich et al., 2005; Eagleman, 2009). In semantic dementia (SD), the processing of ordinal stimuli appears to be preserved relative to non-ordinal ones (Cappelletti et al., 2001). Moreover, ordinal stimuli often map onto unconscious spatial representations, as observed in the SNARC effect (Dehaene et al., 1993; Fias, 1996). At present, little is known about the neural representation of ordinal categories. Using functional neuroimaging, we show that words in ordinal categories are processed in a fronto-temporo-parietal network biased toward the right hemisphere. This differs from words in non-ordinal categories (such as names of furniture, animals, cars, and fruit), which show an expected bias toward the left hemisphere. Further, we find that increased predictability of stimulus order correlates with smaller regions of BOLD activation, a phenomenon we term prediction suppression. Our results provide new insights into the processing of ordinal stimuli, and suggest a new anatomical framework for understanding the patterns seen in synesthesia, unconscious spatial representation, and SD.

## Introduction

Overlearned ordinal categories, whose members carry an inherent sequence to them (e.g., days of the week, months of the year, letters of the alphabet, or the integer numbers), appear to belong to a special class of stimuli. One indication comes from synesthesia, a perceptual condition in which a perceptual experience, such as color, is triggered by an unrelated sensory input (Cytowic and Eagleman, 2009). Most synesthetic experiences are triggered by members of learned ordinal categories such as letters, numbers, days of the week, and months of the year (Rich et al., 2005; Eagleman, 2009).

Another indicator of the uniqueness of ordinal categories comes from semantic dementia (SD), in which numerical stimuli are often preserved while processing of non-ordinal categories (e.g., names of animals, furniture, fruit, and cars) is impaired (Cappelletti et al., 2001; Halpern et al., 2004). Although there has not been a detailed characterization of ordinal category-processing in a large sample of SD patients, there is some evidence to suggest relatively intact processing of other ordinal categories in SD (Cappelletti et al., 2001).

A third indication of the special status of ordinal categories comes from the finding that they can acquire a spatial representation that influences the allocation of spatial attention. In cultures that read numbers and words from left to right, individuals are quicker to react to members later in an overlearned category (e.g., the second half of the alphabet) when the stimuli are presented in the right (or top) half of visual space, an effect known as the Spatial-Numerical Association of Response Codes (SNARC) effect (Dehaene et al., 1993; Fias, 1996). SNARC effects have been observed with non-numerical ordinal stimuli such as letters, days of the week, and months of the year (Gevers et al., 2003, 2004).

Collectively, the above observations suggest a different encoding for ordinal vs. non-ordinal stimuli in the brain. To test the hypothesis that stimuli from ordinal categories are processed differently than stimuli from non-ordinal categories, we had participants carry out a semantic task while their neural activity was measured with functional magnetic resonance imaging (fMRI). Further, we explored whether the neural correlates of ordinality speak to the behavioral consequences of predictability that stem from the order of presentation of ordinal stimuli.

## Materials and methods

### Participants

Forty-one participants (16 female; mean age range = 23.9; all right handed) with normal or corrected-to-normal vision participated in the experiment after giving written consent in accordance with the Institutional Review Board at Baylor College of Medicine. Six participants were removed from analysis due to realignment failure during pre-processing.

### Experimental procedure

**Figure 1:**
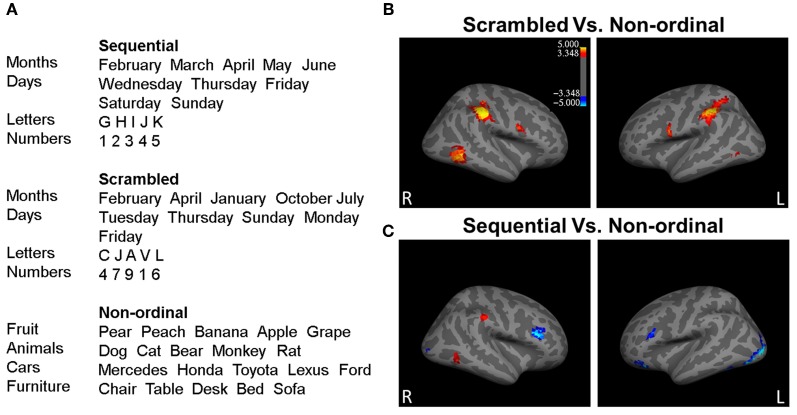
**Processing of ordinal stimuli involves more right hemisphere processing**. **(A)** Example stimuli presented during the experiment from each of the three stimuli categories. **(B)** The right middle temporal gyrus (rMTG), the right inferior parietal lobe (rIP) including right supramarginal gyrus (rSMG), the left inferior parietal lobe (lIPL), the left inferior frontal gyrus/ventral precentral gyrus (lIFG), and the right inferior frontal gyrus/ventral precentral gyrus (rIFG) show greater activity to *Scrambled* stimuli (red; *p* < 0.05 corrected for multiple comparisons). **(C)** The rSMG, rMTG, and the lIPL display greater activity for *Sequential* trials, while the left occipital lobe extending into the inferior temporal lobe, the left and right inferior frontal gyrus, the right occipital lobe, and the left middle frontal gyrus bilateral inferior parietal lobes, the right angular gyrus, the rMTG, and the right medial prefrontal cortex (rmPFC) respond with greater activity to *Non-ordinal* stimuli (blue; *p* < 0.05, corrected for multiple comparisons). *n* = 35.

Participants performed a simple oddball task while in the MRI scanner. Each trial in the experiment consisted of 5 words that were presented serially for 500 ms each with interstimulus intervals of 300 ms. Randomly interleaved trials represented one of three conditions (Figure 1A): (1) words in an ordinal category were presented in their proper order (*Sequential condition*), (2) words in an ordinal category were presented in a scrambled order (*Scrambled condition*), or (3) words belonged to a non-ordinal category (*Non-ordinal condition*). To ensure that participants remained attentive inside the scanner, on 50% of the trials the fifth stimulus would be an oddball (e.g., “Monday, Tuesday, Wednesday, Thursday, Banana”, or “Pear, Peach, Grape, Apple, 8”). Six to ten seconds after the last stimulus, a question appeared on the screen: “Was there an oddball?” Participants made a “yes” or “no” response using a button box, and the next trial commenced 6–10 s later. Participants completed 20 practice trials outside the MRI scanner and 120 trials in the scanner. All 120 experimental trials were carried out in one functional run, lasting approximately 45 min.

Words were presented in black font on a light background (~20 cd/m^2^). Average lengths of words in ordinal and non-ordinal categories were 2.9 and 4.9 letters, respectively. The ordinal and non-ordinal items were not explicitly matched on age of acquisition or familiarity but were comparable on usage frequency (Table A1). Each word subtended on average a visual angle of ~1.5°.

### fMRI data acquisition and pre-processing

High-resolution T1-weighted anatomical scans were acquired on a Siemens 3.0 Tesla Allegra scanner using an MPRage sequence. Functional run details were as follows: echo-planar imaging (EPI), gradient recalled echo; repetition time (TR) = 2000 ms; echo time (TE) = 40 ms; flip angle = 90°; 64 × 64 matrix, 26 4 mm axial slices, yielding functional 3.4 × 3.4 × 4.0 mm voxels. Parts of the cerebellum were excluded from the slices.

### Data analysis

Data preprocessing and analysis were performed using SPM8 (http://www.fil.ion.ucl.ac.uk/spm/software/spm8) and the ART toolbox (http://www.nitrc.org/projects/artifact_detect/). Additionally, the AFNI program 3dClustSim was used to obtain threshold information (http://afni.nimh.nih.gov/pub/dist/doc/program_help/3dClustSim.html). Images were created using FreeSurfer (http://surfer.nmr.mgh.harvard.edu/).

Motion correction was carried out by co-registering data to a base volume. TRs in which head motion exceeded a cutoff (1 mm of translation or rotation between consecutive TRs) were removed. TRs that were outliers (2 standard deviations away from mean) in global brain activation were omitted from further analysis as well.

The average of the motion-corrected images was co-registered to each individual's structural MRI using a 12 parameter affine transformation. EPI images were spatially normalized to the MNI template (3.4 × 3.4 × 4 mm voxels) by applying a 12 parameter affine transformation, followed by a non-linear warping using basis functions (Kao et al., 2005). Images were then smoothed with a 6 mm isotropic Gaussian kernel and highpass filtered in the temporal domain (filter width of 128 s).

To identify regions involved in processing ordinal stimuli, we performed a general linear model (GLM) regression. Regressors were defined from the onset times of Sequential trials, Scrambled trials, and Non-ordinal trials. Oddball trials for each of these conditions were defined as separate regressors in the GLM, but as they confounded predictability, they were excluded from further analysis for the purposes of this paper. Additionally, the timing of subjects' button presses and head movement parameters were included in the GLM as effects of no interest. In total, there were 14 types of events in the GLM. The events were convolved with a canonical hemodynamic response function to create the regressors used for analysis. After performing the regressions, we formed three random-effects contrasts. All *p*-values were corrected for multiple comparisons using an uncorrected *p*-value of 0.001 and a cluster size threshold of 11 voxels to obtain a corrected *p* < 0.05 (3dClustSim; Forman et al., 1995).

## Results

In the scanner, participants performed the oddball detection task with an average accuracy of 96.53%, indicating appropriate attentiveness. There was no significant difference between participants' performance on ordinal and non-ordinal trials for oddball detection accuracy (paired *t*-test; *p* = 0.3). Trials which included oddball stimuli were not included in the present analysis.

To determine which regions responded to ordinal stimuli—regardless of the order of presentation—we contrasted Scrambled trials over Non-ordinal trials (Figure 1B; Table A2). This revealed greater activity in the right middle temporal gyrus (rMTG), the right inferior parietal lobe (rIP) including right supramarginal gyrus (rSMG), the left inferior parietal lobe (lIPL), the left inferior frontal gyrus/ventral precentral gyrus (lIFG), and the right inferior frontal gyrus/ventral precentral gyrus (rIFG) in response to Scrambled trials. There were no significant clusters that displayed greater activation to Non-ordinal trials (*p* < 0.05 corrected for multiple comparisons, random effects analysis).

Next, to determine the effect of predictability in the order of presentation, we compared Sequential trials and Non-ordinal trials (Figure 1C; Table A2). This contrast revealed greater activation in the right supramarginal gyrus (rSMG), rMTG, and the lIPL for Sequential trials. In contrast, the Non-ordinal condition induced greater activity in the left occipital lobe extending into the inferior temporal lobe, the left and right inferior frontal gyrus, the right occipital lobe, and the left middle frontal gyrus (*p* < 0.05 corrected for multiple comparisons, random effects analysis).

**Figure 2:**
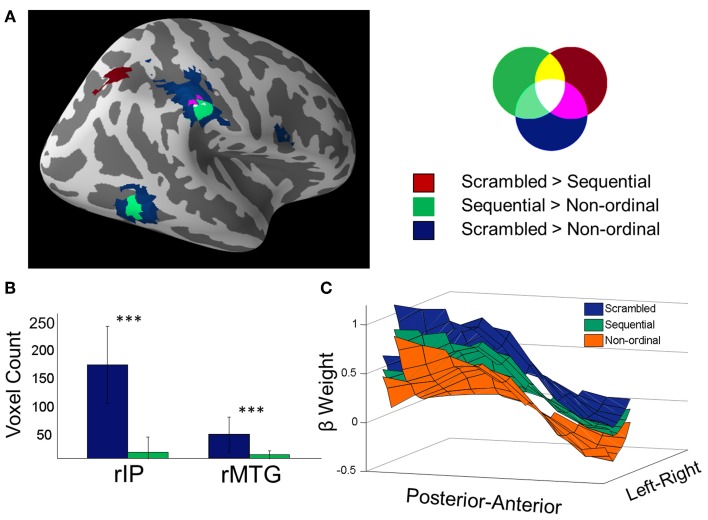
**Prediction suppression: *Scrambled* stimuli recruit greater activity in temporo-parietal networks than *Sequential* stimuli**. **(A)** Overlay of Scrambled > Non-ordinal (blue), Sequential > Non-ordinal (green), and Scrambled > Sequential (red) contrasts shown in Figures 1B and C (*p* < 0.05 corrected). **(B)** Voxel counts of the clusters from the rIP and the rMTG from the previous two contrasts. In order to obtain a value subjectable to statistics, the contrasts were performed 30 times, each time using 70% of subjects (25 out of 35) (a bootstrapped voxel count). The resulting comparison shows that *Scrambled* stimuli recruit greater volumes than *Sequential* stimuli in the MTG and rIP (^***^p < 0.001, repeated measures *t*-test). **(C)** Beta weights in the rIP are shown here averaged across the superior-inferior axis (z-axis) for all three conditions (for visualization only). The mask includes voxels that were found from either the contrast of Scrambled trials over non-ordinal trials, the Sequential over non-ordinal trials, or Scrambled over Sequential trials. Both amplitude and spatial extent of the rIP cluster decrease when ordinal stimuli are presented in a predictable order, as compared to a scrambled order.

To identify regions that were involved in processing ordinal stimuli whether or not they were presented in their natural (or predictable) order, we next focused on the conjunction of the above two contrasts (Nichols et al., 2005). The regions significantly activated in *both* the Sequential > Non-ordinal and Scrambled > Non-ordinal contrasts are shown in Figure 2A. The conjunction reveals three regions that display greater activity in response to members of ordinal categories regardless of their order of presentation—the rSMG (23 voxels), the rMTG (18 voxels), and the lIPL (15 voxels; *p* < 0.05 corrected for multiple comparisons, random effects analysis).

Next, to directly assess the effect of predictability, we performed a whole-brain contrast of Scrambled trials over Sequential trials (Figure 2A, red). This yielded a cluster within the rIP and the right superior parietal lobe; there were no significant clusters in the reverse contrast.

**Figure A1:**
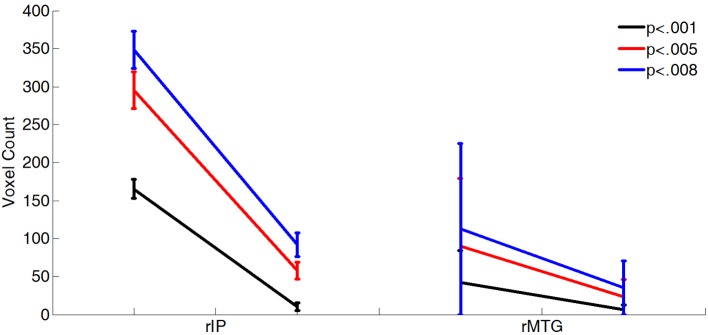
**The difference in the sizes of activated clusters is not an artifact of the chosen statistical threshold**. More voxels were activated in the rMTG and rIP in *Scrambled* trials than in the *Sequential* trials relative to the *Non-ordinal trials* at several different thresholds. Eighty-three percentage of subjects were randomly selected for 30 iterations of each contrast. All differences between sequential and scrambled clusters are significant by paired *t*-test (*p* < 0.001).

In both the rIP and the rMTG, we found that the response to Sequential stimuli spans a smaller volume than the response to Scrambled stimuli. A bootstrapped voxel count in these two regions (30 iterations of 70% of subjects, uncorrected *p* < 0.001, no cluster size restriction) found the number of voxels in the *Scrambled trials > Non-ordinal* contrast (rIP, 165 ± 68 voxels; rMTG, 42 ± 31 voxels) to be significantly greater than the number of voxels in the *Sequential > Non-ordinal* contrast (Figure 2B; rIP, 11 ± 27 voxels; rMTG, 6 ± 7 voxels; repeated measures *t*-test, rIP, *t*_(29)_ = 12.36; rMTG, *t*_(29)_ = 6.29; *p* < 0.001). This change in cluster size is not a result of the statistical threshold, as this effect is maintained at a variety of thresholds (Figure A1). Note that although we did not find a significant cluster within the rMTG in our Scrambled > Sequential contrast, a more liberal threshold (uncorrected *p* < 0.005) revealed increased activation in Scrambled relative to Sequential conditions here. Taken together, our results suggest increased efficiency with increasing predictability in the rIP (Figure 2C), and potentially in the rMTG as well.

**Figure 3:**
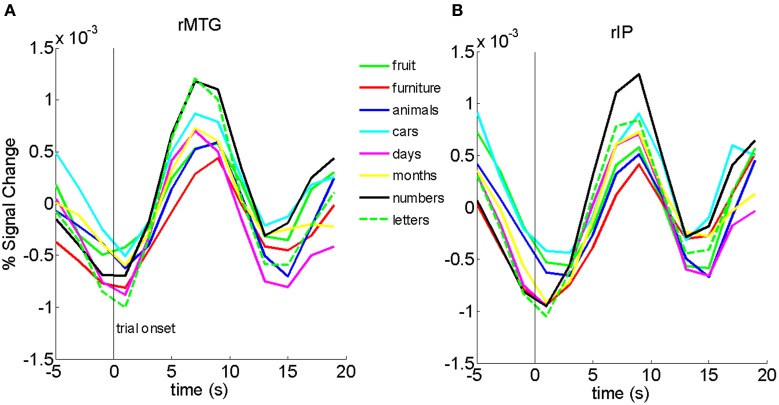
**Bold traces in the rMTG (A) and rIP (B) show that no one particular stimulus appears to drive the results in Figure 1**.

Finally, to ensure that the differences we found between ordinal and non-ordinal stimuli were not driven mainly by one type of stimulus in particular (e.g., numbers or letters of the alphabet), we analyzed the time-series data for the eight different types of stimuli in the Sequential and Non-ordinal conditions for the rIP and rMTG (Figure 3). Qualitatively, the activity in the right middle temporal gyrus and the right inferior parietal lobe does not appear to be driven by any one stimulus in particular. Because there were a small number of trials per sub-type of stimulus, we lack sufficient power to carry out a more rigorous exploration of this question.

## Discussion

Although semantic processing is thought to predominantly engage the left hemisphere (Binder et al., 1997), we have found that the processing of ordinal stimuli involves more right hemisphere activation, specifically in the right middle temporal gyrus and the right inferior parietal lobe.

SD typically involves extensive atrophy of the left (dominant) temporal lobe (Chan et al., 2001). Our results may thus serve to explain why the processing of ordinal stimuli is selectively preserved in SD (Cappelletti et al., 2001; Grossman and Ash, 2004; Halpern et al., 2004), as well as in aphasias resulting from lesions to the left temporal cortex (Thioux et al., 1998; Varley et al., 2005). That is, even while patients lose the capacity to generate words, they can still recite sequences such as numbers and days of the week. Currently, it is difficult to dissociate the effect of “overlearnedness” or familiarity from the effect of belonging to an ordinal category. What is important for our purpose is the fact that these ordinal elements (weekdays, months, letters, numbers) appear to shift to a preferentially right hemispheric processing, where they can be spared from left hemisphere damage.

It is possible that our results can be explained by the fact that our ordinal stimuli are slightly more abstract, with concurrent low imageability or ability to visualize, while our non-ordinal stimuli are more concrete (Table A1). However, weighing against this possibility is the general observation that more left hemisphere activation is seen in response to abstract concepts over concrete ones (Sabsevitz et al., 2005). Because our ordinal stimuli involve more right hemisphere activation than non-ordinal stimuli, the abstractness explanation of our data is not strongly supported. Further, although the right temporal lobe (including the MTG) has previously been implicated in networks involved in the processing of abstract stimuli (Sabsevitz et al., 2005), the rIP has not. This leads us to suggest that our findings reveal something about ordinality beyond mere abstractness.

Members of ordinal categories carry a rank within the set. As such, presenting them in their natural order could lead to strong expectations about what is to follow, and these effects on predictability can shrink the perceived duration of sequential stimuli (Pariyadath and Eagleman, 2007, in press). Here, we found that the amplitude and spatial extent of neural activation diminishes in rIP, and possibly in the rMTG, when ordinal stimuli are presented in their natural order (Figure 2). In other words, increasing predictability results in a more efficient neural representation of stimuli. Previous research has suggested an attenuation of neural response when perceptual expectations based on very recent events (within the preceding 1–2 min) are fulfilled (Summerfield et al., 2008). To our knowledge, ours is the first piece of evidence indicating that long-term experience drives similar expectation-related attenuation of neural response. Collectively, we summarize our findings and those of Summerfield et al. (2008) under the term “prediction suppression,” an analog to repetition suppression. Further, because there is decreased neural activation in conditions that typically result in decreased perceived duration (Pariyadath and Eagleman, 2007), our current data support the hypothesis that subjective duration is linked to efficiency of neural representation (Eagleman and Pariyadath, 2009).

Previous studies have implicated the rIP in time perception (Rao et al., 2001), and one model posits that the inferior parietal cortex is the heart of a common magnitude system, one in which computations of space, time, and quantity are based (Walsh, 2003). Here, we have shown that when stimulus presentation order becomes predictable (by virtue of position in an overlearned sequence), the amplitude and spatial extent of activation within the rIP decreases. As mentioned in the last paragraph, the predictability of a stimulus influences its perceived duration (Pariyadath and Eagleman, 2007, in press). It is reasonable to infer now that the interaction of predictability and duration may be mediated by the rIP. More studies are needed to elucidate the mechanisms by which increasing predictability might translate into decreased activation in regions involved in processing time specifically and magnitude in general.

The observation that synesthesia typically involves the triggering of a sensory experience by elements of ordinal categories (Rich et al., 2005; Cytowic and Eagleman, 2009; Eagleman, 2009) suggests that synesthetes might show greater functional or structural connectivity between color regions and the right hemisphere areas described here (such as MTG). Indeed, studies in synesthetes find increased BOLD activation in the right MTG and increased structural connectivity in the nearby right inferior temporal gyrus (Rouw and Scholte, 2007). In this same vein, new studies demonstrate increased white matter integrity in the right-hemisphere inferior fronto-occipital fasciculus (a tract which includes white matter underlying the right MTG; Zamm et al., under review), further supporting the hypothesis of increased connectivity in synesthetes between color regions and regions involved in overlearned sequences.

One possible framework for our results is that the relative position (i.e., location) of an item in an ordinal category is a salient feature of its representation—specifically, that positions within ordinal categories are analogous to positions in space. This hypothesis would make our results consonant with the finding that the right hemisphere is more involved in the processing of elements with coordinates (elements in specific locations), while the left hemisphere is more concerned with categorical relationships (e.g., inside/outside, above/below) (for a review, see Jager and Postma, 2003). The hypothesis that ordinal sequences are encoded by analogy to spatial locations is also consistent with the SNARC effect, which unmasks an implicit spatial-coordinate representation of the number lines, alphabets, weekdays, and months (for review, see Hubbard et al., 2005). Further, in spatial sequence synesthesia, the relationship between ordinality and space is perceptually explicit: sequences such as weekdays, months, letters, and numbers are experienced as having specific locations in relation to one another (Cytowic and Eagleman, 2009; Eagleman, 2009).

Given the above observations, our data offer a new prediction: if participants were to be overtrained on two new sets of arbitrary symbols—one taught as a ordinal category and one as a non-ordinal category—we may be able to witness the transfer of the encoding of the ordinal set (but not the non-ordinal set) to the right hemisphere with learning. This is a subject of current investigation in our laboratory. An open question is whether the right-lateralized processing is unique to ordinal stimuli learned during childhood, or instead whether similar activation can be reproduced in brains of adults who are overtrained on new ordinal categories. We are also testing whether, in synesthesia, such a transfer corresponds in time to a new ordinal category which begins to trigger color experiences.

### Conflict of interest statement

The authors declare that the research was conducted in the absence of any commercial or financial relationships that could be construed as a potential conflict of interest.

## Appendix

### The neural representation of overlearned sequences

**Table A1:** **Age of acquisition, usage frequency, and imageability of stimuli used in the experiment**.

**Category**	**Age of acquisition^*^ (months)**	**Usage frequency^†^ (per million words)**	**Imageability^‡^ (scale: 1 = poor, 7 = high)**
Animals	32	18.96	6.29
Fruits	42.4	5.67	6.71
Furniture	34.52	96.43	6
cars	–	–	4.86
Numbers	42	–	4.71
Letters	42	–	3.86
Days	48	40	2.86
Months	48	43.42	3

* *Morrison et al., 1997; Cytowic and Eagleman, 2009*.

† Usage frequency was obtained from the COBUILD corpus, which was accessed via the WebCelex website. http://www.mpi.nl/world/celex

‡ *Imageability ratings were obtained from seven naive participants who rated the stimuli used in the experiment on a 7-point scale (where 1 indicated poor imageability and 7 indicated high imageability)*.

**Table A2:** **Brain areas activated during the different experimental conditions**.

**Area (Hemisphere)**	**Brodmann area**	**MNI coordinates at peak activation**			**Maximum *t*-statistic**	**Cluster size (No. of voxels)**
**SCRAMBLED TRIALS > NON-ORDINAL TRIALS**						
Temporal Lobe (R) including the middle temporal gyrus and the inferior temporal gyrus	19/21/37	58	−57.6	−6	5.25	80
Temporal Lobe(L) including the middle temporal gyrus and the inferior temporal gyrus	37	−50.8	−67.8	−2	3.96	23
Frontal Lobe (L) including the inferior frontal gyrus and precentral gyrus	44	−50.8	3.6	18	4.45	25
Frontal Lobe (R) including the inferior frontal gyrus and precentral gyrus	44	51.2	7	22	4.43	25
Parietal Lobe (L) including the inferior parietal lobe, the supramarginal gyrus, and the postcentral gyrus	40/2	−50.8	−40.6	50	5.42	239
Parietal Lobe (R) including the inferior parietal lobe, supramarginal gyrus, and postcentral gyrus	2/3/40	47.8	−33.8	46	6.35	268
**SEQUENTIAL TRIALS > NON-ORDINAL TRIALS**						
Temporal Lobe (R) including the middle and inferior temporal gyri	19/37	54.6	−61	−6	4.58	18
Parietal Lobe (R) including the angular gyrus, the supramarginal gyrus, and the inferior parietal lobe	40	54.6	−54.2	34	3.79	13
Parietal Lobe (L) including the inferior parietal lobe and the supramarginal gyrus	40	−64.4	−37.2	30	4.39	17
Parietal Lobe (R) including the supramarginal gyrus and the inferior parietal lobe	2/40	58	−27	42	4.00	25
**SEQUENTIAL TRIALS** < **NON-ORDINAL TRIALS**						
Occipital and Temporal lobes (L) including the fusiform gyrus, the middle and inferior occipital gyri, the lingual gyrus, and the parahippocampal gyrus	18/19/36/37	−23.6	−91.6	−6	−8.50	157
Frontal Lobe (R) including the inferior and middle frontal gyri	11/47	−30.4	30.8	−14	−5.36	20
Occipital Lobe (R) including the lingual gyrus, and the middle and inferior occipital gyri	18	20.6	−91.6	−2	−6.05	52
Frontal Lobe (L) including the inferior frontal gyrus and the mid-frontal gyrus	47	−30.4	30.8	−14	−5.92	38
Frontal Lobe (L) including the inferior frontal gyrus	46	−40.6	20.6	22	−4.32	19
Frontal Lobe (R) including the middle and inferior frontal gyri	46	44.4	27.4	18	−5.33	47
**SCRAMBLED TRIALS > SEQUENTIAL TRIALS**						
Parietal Lobe (R) including the superior parietal lobe	7	30.8	−61	46	3.66	12
Parietal Lobe (R) including the supramarginal and postcentral gyrus	2/40	54.6	−30.4	46	4.11	18
